# Electrocardiographic imaging in the atria

**DOI:** 10.1007/s11517-022-02709-7

**Published:** 2022-11-12

**Authors:** Ismael Hernández-Romero, Rubén Molero, Carlos Fambuena-Santos, Clara Herrero-Martín, Andreu M. Climent, María S. Guillem

**Affiliations:** ITACA, Universitat Politècnica de València, Valencia, Spain

**Keywords:** Electrocardiographic imaging, Atrial arrhythmias, Inverse solution, Catheter ablation, Treatment guidance

## Abstract

The inverse problem of electrocardiography or electrocardiographic imaging (ECGI) is a technique for reconstructing electrical information about cardiac surfaces from noninvasive or non-contact recordings. ECGI has been used to characterize atrial and ventricular arrhythmias. Although it is a technology with years of progress, its development to characterize atrial arrhythmias is challenging. Complications can arise when trying to describe the atrial mechanisms that lead to abnormal propagation patterns, premature or tachycardic beats, and reentrant arrhythmias. This review addresses the various ECGI methodologies, regularization methods, and post-processing techniques used in the atria, as well as the context in which they are used. The current advantages and limitations of ECGI in the fields of research and clinical diagnosis of atrial arrhythmias are outlined. In addition, areas where ECGI efforts should be concentrated to address the associated unsatisfied needs from the atrial perspective are discussed.

**Graphical abstract:**

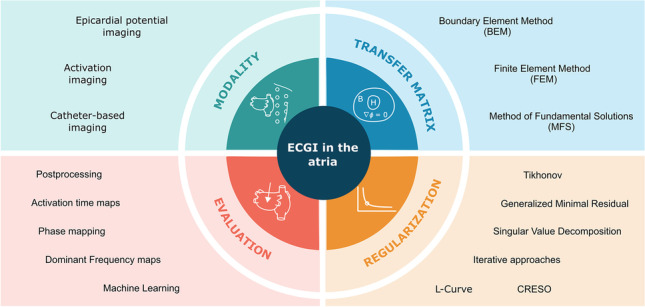

## Introduction

Atrial arrhythmias are common causes of morbidity [1]; although rarely fatal, in patients with various cardiovascular risk factors, structural heart disease, and other comorbidities, they can potentially increase mortality [1–3]. Even though bradyarrhythmias and tachyarrhythmias are the most frequent arrhythmic events in the atria [4], atrial fibrillation occurs as a consequence of several established cardiovascular risk factors [1, 5]. The electrocardiographic diagnostic method for these disorders is the evaluation of the P wave as a sign of atrial activation on the surface electrocardiogram (ECG) [6]. The existence or absence of P waves, their shape, and their occurrence can all be used to detect atrial disorders [1].

While the use of invasive electrophysiological techniques to assess atrial arrhythmias [7, 8] has yielded a mechanistic understanding of the origin of arrhythmias in general, it is now widely accepted that diagnosis with noninvasive techniques such as electrocardiography is sufficient in most cases [9]. However, when there are diagnostic uncertainties or as a prelude to a catheter ablation therapy, atrial mapping by invasive studies may be advantageous [10]. With the increasing trend of the use of catheter ablation procedures to treat atrial arrhythmias [8], research around P waves brings more attention to unmasking the mechanisms of focal and reentrant atrial rhythm disorders on the surface ECG.

In this sense, the inverse problem of electrocardiography or electrocardiographic imaging (ECGI) can provide an extra value as an estimation of the electrical information over cardiac surfaces from non-contact recordings [11]. ECGI has been used to determine the propagation patterns during premature beats or tachyarrhythmias as well as drivers related to reentrant arrhythmias [12].

Recent studies reviewing the status of ECGI have focused on highlighting best practices for validating solutions [13] or characterization of atrial fibrillation using ECGI [14]. The purpose of this review is to technically address the major atrial ECGI studies of recent years and, therefore, to emphasize those areas in which technical development of ECGI solutions has the potential to improve atrial characterization and treatment and to help establish their clinical utility. Thus, the objectives of this work are:

1. To summarize the multiple ECGI methodologies, regularization methods, and post-processing techniques, as well as the circumstances under which they were used in the atria.
2. To highlight the present advantages and limitations of current ECGI applied to research and clinical diagnosis of atrial arrhythmias.
3. To identify the areas in which ECGI research should concentrate efforts in the future in order to take on the unmet needs from the approach in the atrial context.

**Fig. 1:**
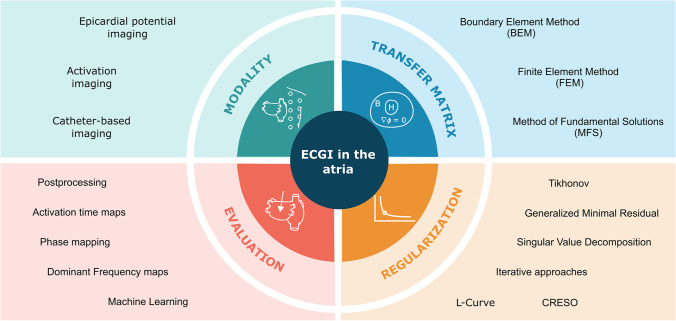
Summary of topics covered in the review

The main sections and concepts on which this review is focused are depicted in Fig. 1. We introduce the different alterations that occur in the atria leading to atrial arrhythmias, the approaches for the use of ECGI, methodological aspects for ECGI, post-processing including alternative approaches to ECGI resolution, and future directions in the use of ECGI to characterize atrial arrhythmias.

## Atrial arrhythmias

Alterations from normal sinus rhythm (SR) are known as tachyarrhythmias. According to their origin and mechanism behind their maintenance, these pathologies can be divided into different groups. The most common supraventricular tachyarrhythmias (SVTs) are atrial fibrillation (AF), atrial flutter (AFL), and atrial tachycardias (ATs) [15].

Atrial fibrillation is the most common type of arrhythmia, with an atrial rate between 300 and 600 bpm [16]. Over the last decades, different theories have been proposed as the ones responsible for its initiation and maintenance [17–19]. They are mainly divided into three (not mutually exclusive) theories: ectopic foci (areas that suddenly depolarize leading to a centrifugal wavefront expansion from that point to the rest of the atria) [20], rotors (reentrant patterns of activation which circulate around a region) [21] and multiple unstable waves which propagate through the atria [22]. Moreover, during the last years, two novel mechanisms have been proposed. The first one argued that the presence of rotors might be linked to fibrotic areas and their borders [23] whereas the second one suggests that epicardial-endocardial dissociation is the one which plays a main role in AF maintenance [24].

Atrial flutters are a type of SVTs maintained by a macro reentrant circuit in the heart around either anatomical or functional obstacles and with an atrial rate between 240 and 350 bpm [25]. Depending on the structures involved in this reentry, we can distinguish between typical and atypical flutter. Typical flutter is the most frequent AFL, and it is characterized by a rotational wavefront pattern that pivots around the tricuspid annulus including in its lower part of the circuit, the cavotricuspid isthmus (CTI). Considering the direction of the rotation, we can differentiate between counterclockwise (the most common one) and clockwise typical AFL [6, 26, 27]. On the other hand, atypical AFL involves other reentrant circuits either in the left or right atrium [27, 28].

Atrial tachycardias are characterized by a rapid atrial rate which oscillates between 100 and 250 bpm [6]. They are sustained by an ectopic focus in the atria which produces a centrifugal wavefront expansion from that point to the rest of the atria. These sudden depolarizations are either caused by automatic trigger activity or by the presence of micro-reentries [29, 30]. Moreover, it is also possible that ATs are maintained by various foci leading in this case to multifocal AT [31].

Lastly, there are other less common SVTs related specifically to the sinus node. These are inappropriate sinus tachycardia and sinoatrial nodal reentrant tachycardia. Both are characterized by a fast sinus rate even in a relaxed condition. Regarding inappropriate sinus tachycardia, different mechanisms have been proposed for this pathology from internal problems in the sinus node to dysfunctions in the autonomic or neurohormonal control system. Nevertheless, experts have not achieved any consensus about it yet [32]. Contrary, the sinoatrial nodal reentrant tachycardia is led by micro reentrant pathways around the sinus node, which lead to a focal AT in this area [33]. Some features that differentiate it from sinus tachycardia are that it is characterized by an unexpected onset and termination and that the interval between activations is typically longer than during SR [6].

## Modalities of inverse problem resolutions in the atria

The classical inverse solution can be seen as an extension of the conventional ECG [34], based on surface measurements as inputs to determine the electrical activity on the surface of the heart. However, there are numerous approaches including different source models or the incorporation of physiological constraints into the formulation [35].

**Fig. 2:**
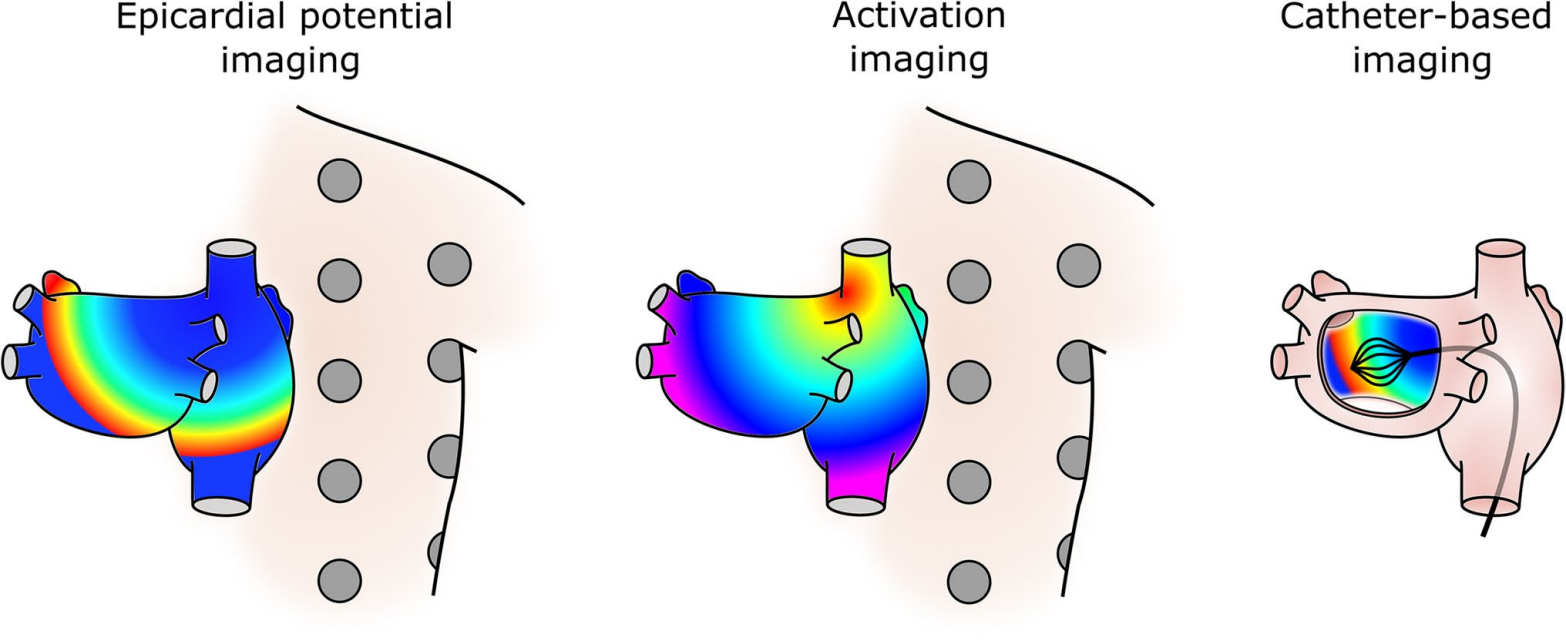
Modalities of inverse problem resolutions in the atria. Reconstruction of epicardial potentials or activation times is accomplished through body surface measurements in the first two scenarios; reconstruction of surface potentials is accomplished in the third case through intracavitary potentials

In Fig. 2, three modalities of inverse solutions in the atria which will be introduced in this section are represented. Depending on the source and the forward model used, body surface potential recordings can be used to reconstruct time series of epicardial surface potentials, inverse computed electrograms (iEGM) [36], or specific electrophysiological parameters such as activation times [37]. The initial setup to obtain these solutions would consist of a dense electrode array used to obtain multiple Body Surface Potential Maps (BSPM) [38], the location of the recording electrodes, and torso and heart geometries that can be obtained using imaging techniques as magnetic resonance imaging (MRI) [39], computed axial tomography (CT) [40] or photogrammetry [41].

Another inverse problem methodology that has been applied in the atria is catheter-based imaging [42]. In this case, it is possible to reconstruct the electrical potential on the inner wall of the chamber using measurements from multielectrode non-contact probes located inside the heart chambers as part of an inverse solution during clinical electrophysiology (EP) procedures [42, 43]. In this configuration, the catheters must be located by the electrical reference electrodes which are connected to the system that will allow tracking positions inside the heart chambers [43]. These probes can also be utilized to reconstruct the endocardial anatomy after a post-processing stage is carried out [44].

Despite the difference in approach between the use of noninvasive recording and non-contact mapping, both techniques aim to characterize atrial mechanisms [14, 45]. Some studies use the noninvasive recording for characterization [46], therapeutic guidance, and in support of ablation therapy [47]. On the other hand, non-contact mapping has been used for the identification and ablation of the arrhythmic substrate in the setting of atrial tachycardias [45].

The disparities between the solutions to these inverse problem modalities are attributable to the source models employed and the different methodological approaches used in each case, which will be described in more detail in the following sections of this article.

## Derivation of transfer matrix

The first step in solving the inverse problem is to define the relationship between the acquired sources and the volumetric conductor model. This framework is referred to as the forward problem of electrocardiography [48]. A description of the heart and body surfaces that separate regions of different conductivity, as well as the tissue conductivities of each of these regions, is required in order to solve this problem successfully [49]. The purpose is to find a matrix of transfer coefficients that relate the cardiac potentials as a linear combination to those on the measured source potentials, either in the body or in the heart [50].

To begin, the volume conductor is defined in this framework as the region between a surface enclosing the cardiac sources and the conductive outer surface of the volume [50]. This definition effectively eliminates all sources from the solution domain, allowing to employ Laplace’s equation:

1 $$\nabla \left(\sigma \nabla \Phi \right)=0$$

where *σ* is the electric conductivity and $\Phi$ is the electric potential [50].

The most frequently used numerical techniques for solving this problem, in this case, are the finite element method (FEM) [51], the boundary element method (BEM) [48], and the method of fundamental solutions (MFS) [52].

BEM and FEM methods are mesh-based methods, as they require the topological relationships between the nodes in order to convert Laplace’s equation to surface integral form with Green’s theorem. BEM formulation is based on surface integrals and boundary potentials are thus discretized and assumed to be constructed as linear combinations of basic functions [50, 53]. FEM is based on approximating the problem by means of small volumetric elements, called finite elements, so that the residual of the discretized solution is minimized at the Dirichlet and Neumann boundary conditions of the complete discretized volume [51, 54].

The main advantage of BEM is that the number of nodes or degrees of freedom involved in the calculation is much smaller than in FEM, since just the surfaces and not the full volume are discretized. A comparative study applying BEM and FEM to ECG problems has revealed that under similar levels of discretization, BEM produces smaller errors and consumes less computational time but requires more memory than FEM [55–57]. In contrast, FEM accounts for the anisotropic conductivities of human bodies more thoroughly.

On the other hand, MFS is meshless [58], as it does not require a complete torso’s geometry, only the location of the electrodes and the potentials are expressed as a linear combination of Laplace’s fundamental solution over a discrete set of virtual source points following Dirichlet’s and Neumann’s boundary conditions in a Cauchy problem.

MFS methodology does not rely on geometry, so negative effects caused by segmentation errors and singularities in the boundaries are avoided. However, BEM and FEM have the advantage of allowing for more precise geometries, which allows for a more accurate calculation of the transfer matrix. This enhancement is not possible in MFS [58].

## Regularization and regularization parameter optimization in atrial signals

Electrocardiographic imaging is a noninvasive technique that requires information from surface recordings and the geometry of the torso and the heart of the patient to estimate cardiac potentials. ECGI resolution is an ill-conditioned problem and, therefore, small perturbations in the data produce significant errors in the inverse solutions obtaining unrealistic results [36]. Regularization is typically used in most approaches for inverse problem resolution in order to overcome the ill-posedness of the problem. During the last decades, different regularization techniques and optimization methods have been proposed [35] and widely studied by multiple research groups in an attempt to solve one of the main challenges of ECGI, the ill-posedness of the inverse problem, which is particularly relevant in the context of atrial arrhythmias because of the low signal to noise ratio present in ECG signals.

One main source for the ill-posedness nature of the ECGI comes from the transfer matrix (*A*) that relates the epicardial potentials and the surface potentials in the forward problem defined in Eq. 2 [35]:

2 $$B=AX+N$$

where *X* are the epicardial potentials, *B* the surface potentials, and *N* the noise. The matrix *A* is generally taken to be temporally invariant and not generally squared and therefore is not invertible [35].

### Tikhonov regularization

To obtain the epicardial potentials (*X*) and minimize the errors in the inverse solution, Tikhonov regularization has been widely used for obtaining noninvasive signals [59], which consists in minimizing the Eq. (3) or a compromise between the least-squares solution and the residual norm of the solution:

3 $$X=\mathrm{min}\left[{\Vert AX-B\Vert }_{2}^{2}+ {{\lambda }^{2}||RX||}_{2}^{2}\right]$$

where *λ* is a regularization parameter and *R* is a squared matrix which can take different formulations and defines the order of Tikhonov regularization. For zero-order Tikhonov, R equals the identity matrix and imposes a limit on the magnitude of the solution. First-order Tikhonov uses the gradient operator for *R*, and second-order Tikhonov uses the surface Laplacian operator, providing a less smooth inverse solution [35]. Since the earliest works on electrocardiographic imaging, Tikhonov regularization has been used and compared with other regularization alternatives, but the vast majority of the studies using atrial signals use this regularization approach [14]. However, other approaches have been applied, like the Generalized Minimal Residual method [60], singular value decomposition [61], iterative approaches [62, 63], and hybrid combinations between Tikhonov-GMRes methods [64].

### Regularization parameter Identification

The optimal regularization would be the one that finds the optimal regularization parameter (*λ*), and this is where more approaches have been studied and tested by other research groups, being the L-curve and CRESO (composite residual and smoothing operator) [65] methods commonly used.

#### L-curve regularization

The L-curve method [66] consists of representing the two main terms of Tikhonov regularization, the least-squares and the residual of the solution, for different values of the regularization parameter in a log–log scale. This plot is L-shaped and allows determining the optimal *λ* at the corner of the curve where these two errors are minimized. This point represents a compromise between an over-regularized and an under-smoothed solution. L-curve optimization, in contrast with other methods, is more robust to changes due to small errors of the signals [67] and has an acceptable computational time. To find the corner of this curve, the point of maximum curvature is typically calculated [67].

#### Composite residual and smoothing operator

On the other hand, the CRESO method [68] consists of finding the smallest value of *λ* that results in a local maximum of the following equation:

4 $$C\left({\lambda }_{t}\right)={\Vert X({\lambda }_{t})\Vert }_{2}^{2}+ {2\lambda }_{t}^{2}\frac{d}{d{\lambda }_{t}}{||X({\lambda }_{t})||}_{2}^{2}$$

These two methods for finding the optimal regularization parameter have been used for Tikhonov regularization indistinctly using FEM [51], BEM [48], and MFS [52] for addressing the relationship between epicardial and surface potentials [48]. BEM and zero-order Tikhonov regularization with the L-curve have shown good results for different types of signals, especially in the context of AF [14]. For instance, in atrial models, it has been proven to be able to reconstruct epicardial activity for detecting dominant frequencies [69, 70] and reentrant patterns [70], later validated in real patients with intracavitary data [41, 71]. Moreover, this regularization has been used as part of the methodology for solving inaccuracies in atrial placement with good results, by minimizing the ECGI reconstruction error [72]. On the other hand, Tikhonov regularization and CRESO optimization have been applied in multiple studies and trials with atrial signals employing CardioInsight’s system, either SR [73], AF [74], or trial tachycardias [75, 76]. Using this approach, Cuculich et al. could distinguish between activation patterns among clinically defined groups of AF patients [46], and Haissaguerre et al. [47] demonstrated the potential of ECGI as a technique for mapping non-invasively AF drivers like unstable rotors and guide ablations with remarkable success. Both approaches, although with limitations, have shown good performance for evaluating the atrial substrate and the power of ECGI as a diagnostic and ablation guidance technology [14].

### Generalized minimal residual method

In addition to the previously described methods, Wang et al. [77] used the Generalized Minimal Residual method (GMRes) [60] to detect the earliest activation sites during atrial tachycardias and successfully guide ablations. GMRes is an iterative approach that belongs to the class of Krylov subspace iterative methods. GMRes has been applied to SR signals to analyze the P waves with proper repolarization starting at the sinus node [78–80]. GMRes has demonstrated its ability to evaluate the epicardial activity and showed that in specific cases works better than the Tikhonov method and the L-curve when the corner of Tikhonov is not easily identified [64]. A combination of GMRes and Tikhonov also showed good results and may be more reliable in specific cases [64]. Despite this fact, there is no theoretical explanation for considering GMRes as a better option for Tikhonov regularization [35, 61].

### Agreement on the best approach

**Fig. 3:**
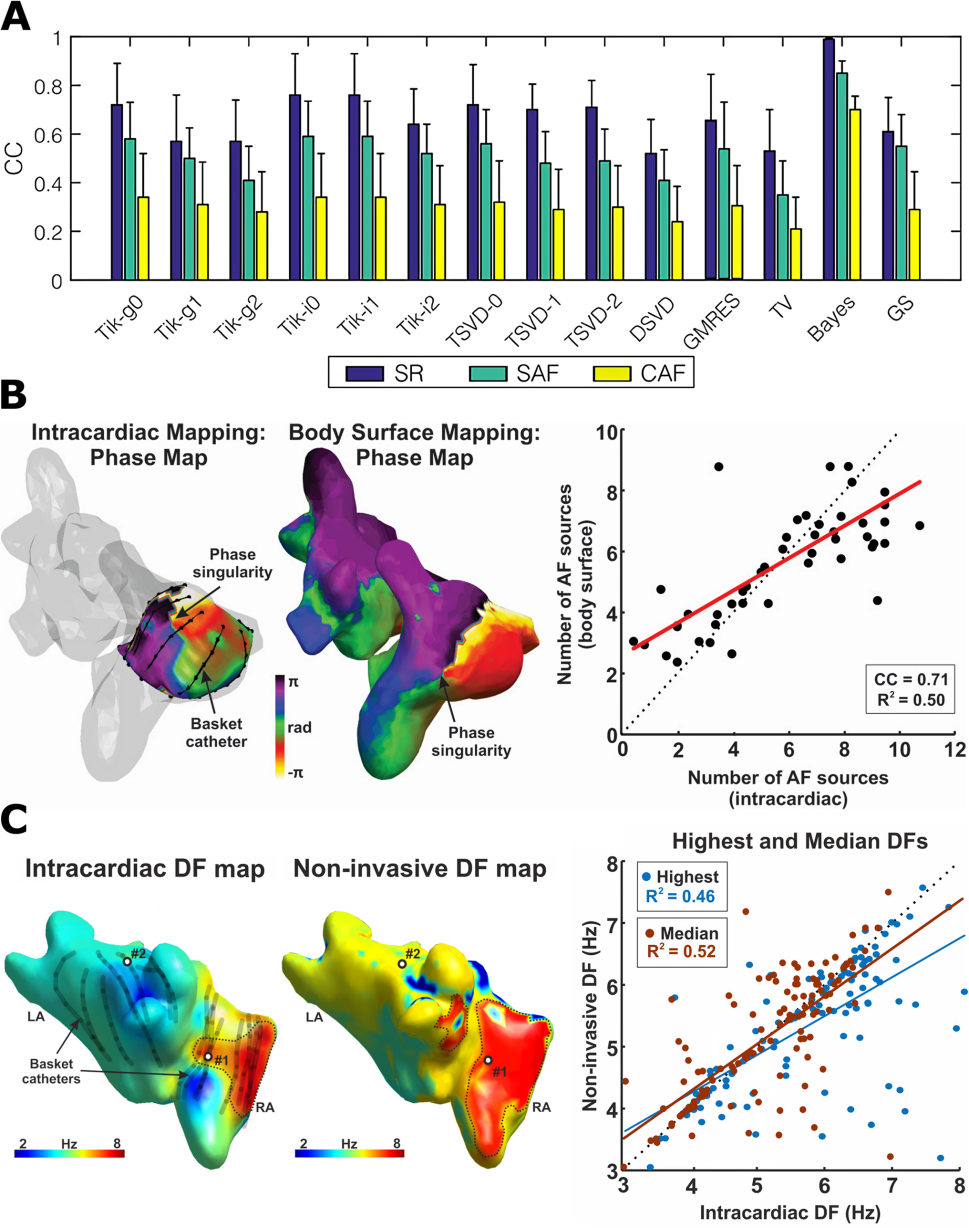
**A** Correlation coefficient of ECGI and simulations of 14 different inverse problem methodologies for sinus rhythm (SR) and simple and complex atrial fibrillation (SAF, CAF), extracted from [61]. **B** Phase singularity of intracardiac and ECGI phase maps and correlation of intracardiac and noninvasive atrial fibrillation sources [71]. **C** Dominant frequency of intracardiac and ECGI maps and correlation of intracardiac and noninvasive dominant frequencies [41]

Several studies have proven the ability of different regularization methodologies to describe the atrial electrical substrate, but there are still open questions about how to minimize the errors when computing the inverse problem. In the context of AF, we compared 14 different methods of regularization for three atrial models of SR, simple and complex AF (Fig. 3A). Bayesian regularization, which makes use of a priori information [81], has been shown to outperform other methods for ECGI resolution, although a priori information is not a real-case scenario in most of the applications [61]. Again in the context of AF, we have shown that the incorporation of the information from intracardiac electrograms (EGMs) may also outperform other ECGI resolution methods [82]. However, if a priori data is not available, zero-order Tikhonov with L-curve optimization was found to be the best approach, even with constant regularization parameters for AF signals. Despite these conclusions, more studies need to address the limitations associated with each type of atrial arrhythmia and not only AF, in combination with a comparison of different regularization and optimization approaches. Moreover, there is a lack of studies on more regularization alternatives as it has been addressed in studies with ventricular signals [63, 83, 84].

### Regularization in non-conventional ECGI

ECGI usually uses surface electrocardiogram recordings to estimate epicardial potentials, but other approaches compute the inverse problem and obtain information on the electrical activity on the surface of the heart.

Activation imaging is an alternative method which provides noninvasive information about the activation times on the epicardium and endocardium instead of the full-time series of epicardial potentials [37]. This problem is nonlinear due to the relationship between the transmembrane potentials of the surface ECG and the activation times. This relationship is solved using the Fredholm integral and BEM formulation. Moreover, this is also an ill-posed problem and, similarly to the conventional ECGI, needs regularization. This regularization substitutes the least-squares error of the Tikhonov regularization with the residual norm of the estimated activation times and the transmembrane potentials of the epicardium. For obtaining the optimal regularization parameter, similar approaches are used as the L-curve. Activation mapping has been validated with intracavitary data with paced atrial signals [85] and atrial flutter [86]. This regularization has only been proven to be accurate for smooth activation patterns, and therefore, its current formulation is not appropriate for complex signals, as in the case of AF.

Alternatively, non-contact charge density mapping consists of using non-contact intracardiac recordings to reconstruct the cardiac geometry and to estimate the value of the charge density in the epicardium by means of the inverse problem resolution [87] and later the epicardial voltage using the forward problem [43]. From the value of the computed charge density on the epicardium, activation times and the time series of potentials can be obtained [42]. Similar regularization approaches are used in conventional ECGI such as Tikhonov regularization or truncated singular value decomposition [87]. Non-contact charge density mapping has been validated in simulations and arrhythmic scenarios for classifying conduction patterns and localized non-pulmonary veins ablation targets in atrial fibrillation [53–56]. This technique can provide real-time anatomical data and signal acquisition with a high spatial resolution, but as opposed to other methods, it is invasive.

## Post-processing in ECGI

The following subsections outline the metrics that are commonly derived from EGMs estimated with ECGI: activation times are used to characterize simple rhythms, such as SR, pacing, and AFL, whereas phase mapping and dominant frequency maps are used in AF.

### Activation time maps

Local activation time (LAT) mapping allows identifying the origin and direction of the electrical propagation, helping therefore in the identification of reentrant circuits, wave breakthroughs, and collisions. This information is presented as a set of isochrones throughout the cardiac tissue indicating the time of activation of each site [66]. Different methods have been developed to estimate activation times in invasive recordings. The most commonly used are those based on the morphology of the signal, like the largest down slope in unipolar electrograms or the center of mass on bipolar electrograms [88].

Obtaining activation times in other atrial rhythms by ECGI has been carried out by different groups. In [85], different pacing sites in the atria were recorded with a 62-channel ECG imaging device. Activation time mapping allowed to locate the exact stimulation location within a 10-mm error in different stimulation areas (the pulmonary veins, coronary sinus, right posterior wall, and high atria). Furthermore, the activation map obtained with ECGI was correlated with CARTO intracavitary data, obtaining a mean correlation of 0.76. Other studies have also demonstrated the feasibility of activation mapping in atrial tachycardias. Z. Zhou et. al. reported successful reconstruction of the activation sequence in 3 patients with typical AFL [89]. The activation maps obtained were also correlated with intracavitary activation mapping obtaining a correlation coefficient of CC = 0.70 ± 0.04. Noninvasive activation mapping in focal tachyarrhythmias was also studied in [75], where this mechanism and origin of the tachycardia could be correctly identified with a higher success rate (21 out of 21 patients presenting this arrhythmia) compared to AFL (23 out of 27 patients). The authors reported higher difficulties associated with the identification of atrial flutters due to the presence of low amplitude P waves.

Most of the traditional methods are oriented to be robust against high fractionated signals with complex morphologies and multiple deflections. However, the main limitation present in ECGI is the loss of sudden transients and sharp morphologies due to the smoothing effect of the inner organs on the projection of cardiac potentials into the body surface [61]. Therefore, the direct application of these algorithms on ECGI signals may result in suboptimal results like false homogeneous regions or artefactual conduction block lines [90]. Similar artefactual block lines have also been reported in the atria during SR in patients with mitral regurgitation (MR) with and without previous history of AF and in healthy volunteers to investigate the physiology of atrial activation [80]. Lines of conduction block were present in 7/9 subjects with AF and MR, in 8/11 subjects with MR without AF, in 8/11 subjects with MR without AF, and in the healthy subject. Despite these artifacts, prolonged left atrial conduction times were found in MR patients that also had AF. In order to overcome this limitation of ECGI-derived activation times, algorithms incorporating spatial correlation to the pure temporal-based estimation have been proposed [91, 92] and could be extended to the study of atrial rhythms.

Regarding atrial fibrillation, additional conditions like a lower signal-to-noise ratio or a more anisotropic conduction system may lead to additional challenges in the estimation of local activation times [93]. These challenges are especially difficult in complex arrhythmias; for this reason, the following subsections are focused on AF.

### Phase mapping

Phase mapping is a common processing technique employed to identify reentries in ECGI [70, 71, 94, 95]. Figure 3B depicts a comparison of phase singularity of intracardiac and ECGI phase maps and the correlation of intracardiac and noninvasive atrial fibrillation sources [71]. Phase maps encode uniquely the different cardiac cycle stages using phase values ranging between − *π* and *π*. In order to calculate unique phases out of potential values, at least two state variables of the electrical propagation dynamics must be tracked. In cardiac simulations, variables like the transmembrane potentials in combination with tissue recovery state have been successfully employed to calculate phases [96]. However, iEGM only represents a single parameter of the electrical state of the heart, the extracellular voltage. In this scenario, two different approaches have been developed to overcome the lack of a second parameter. The first one is the combined use of a voltage signal ($v(x,t)$) and a delayed version of the same ($v(x,t+\uptau )$) to obtain a state representation of the voltage dynamics [97]. The second is the Hilbert transform [98]. This last technique has been widely applied by the research community because it does not require predefining any initial parameter (like delay $\uptau$ in the first approach) [70, 95, 99].

Phase mapping is a very convenient representation to identify reentries in ECGI because it provides a description of the electrical state in the activation cycle without the need to estimate the activation time in iEGMs. Furthermore, in contrast to invasive mapping, where the activation of the heart must be sequentially sampled in small portions of tissue, the panoramic representation provided by ECGI makes it easy to track the movement and main locations with reentries [18]. Reentries are detected by identifying their vortex (or center of rotation). On phase maps, rotor vortices are presented as phase singularities (PSs). These can be identified as points in which no phase value is defined. Nonetheless, the easiest way to detect PSs is by checking the phases in the surroundings of a vortex candidate. If the whole phase range is covered in the surroundings of a particular location, (from − *π* to *π*) then that particular location can be considered a PS [100]. There are multiple implementations to verify the phase distribution around phase singularity points automatically. One of the most popular is the use of the so-called topological charge, where a line integral of the phase change around each location is computed [97]. Specifically, a PS is considered when this integral evaluates to ± 2*π*.

Despite the mentioned advantages, some challenges have been pointed out when dealing with phase mapping in ECGI, mostly when applied to small portions of tissue like the atria or in complex arrhythmias like atrial fibrillation. In the first place, it has been reported that PSs collocate with conduction block areas. The sudden direction changes experienced by wavefront in blocking sites may induce the detection of a PS, even if the wave did not spin an entire turn [94, 101]. Furthermore, other factors like interpolation or far-field effect have been associated with the detection of false positive PS [102, 103]. These were reported in invasive phase mapping reconstructions with a basket catheter, however, to our knowledge, there is very little known about the role of these factors in ECGI. In an in-silico study, we concluded that the presence of several rotors in the atria spinning in opposite directions can lead to the mutual cancelation of the surface signals and, therefore, may prevent rotor identification on ECGI [104]. This is especially true when the rotors are very close together in the atria. Another factor that may reduce the sensitivity of ECGI in rotor detection is the large distance between the electrodes and the atrial substrate, which acts as a natural filter for reentries [18, 104].

**Fig. 4:**
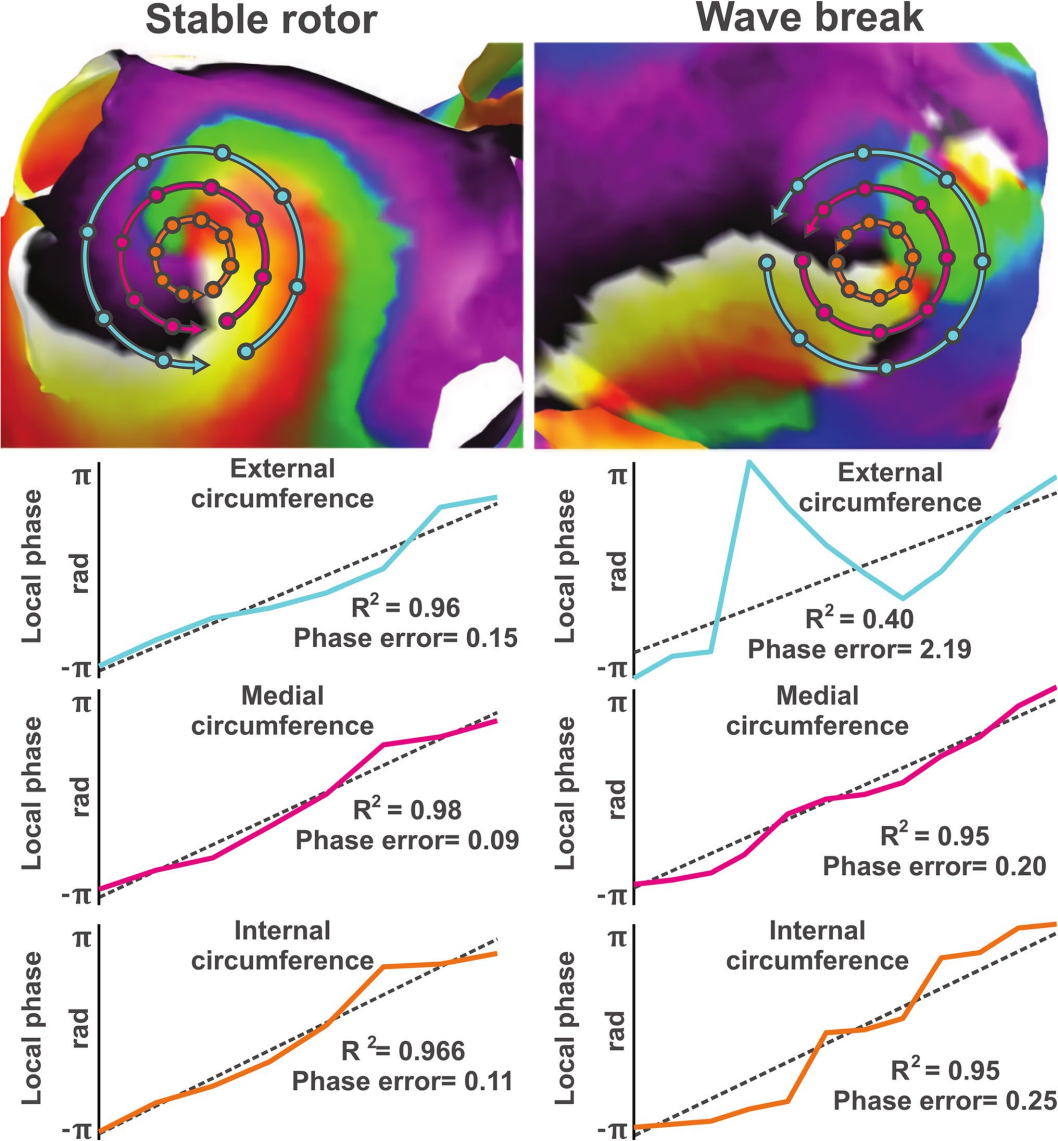
Linearity verification around a PS rotor candidate. Only those PSs with a linear progression of phase in their surrounding should be considered actual reentries [100]

However, reentry detection using phase mapping and ECGI may have some technical limitations. Regarding the possible overestimation of PS due to conduction blocks, we previously presented a methodology to filter out these PSs belonging to non-stable reentries [104]. In this study, we reported that PSs arising from non-reentrant patterns have abrupt phase transitions in their surroundings, while PSs belonging to rotors present an almost linear phase propagation around. This examination was carried out in three circumferences of varying radii around each PSs, as shown in Fig. 4. As a compromise, we proposed to impose a linearity criterion on the phase evolution in the surroundings of each PSs together with a minimum duration of 2 turns in order to avoid the detection of transient propagations that do not constitute a stable reentry.

Another point to consider is how BSPM signal processing affects feature extraction after applying ECGI. For example, it has been shown in ventricles that removing high-frequency noise does not affect the accuracy of electrogram reconstruction but can improve feature extraction after reconstruction [105]. Phase maps can be heavily affected by this issue.

In conclusion, PS detection must be seen carefully and also consider other data sources. ECGI is surely not an exception in this regard. However, the capability of acquiring panoramic views of the entire atria for long periods of time may provide the chance to robustly differentiate those atrial sites driving the arrhythmia from sporadic, artifact-related PS detections.

### Dominant frequency maps

Frequency analysis of inverse computed electrograms becomes especially interesting in the identification of AF drivers. The presence of atrial regions with high-frequency activation rates has been empirically linked to the sustainment of AF in optical mapping [106], and clinical studies [107]. These high-frequency sources are more common in the nearby pulmonary veins, however, they can be present anywhere in the atria [108–110]. This finding makes Dominant Frequency (DF) mapping a necessary tool to understand some AF mechanisms, especially in persistent AF patients.

Regarding the use of DF analysis in ECGI, dominant frequency maps have shown to be a robust representation of the atrial electrical activity in complex arrhythmias like AF. This is because DF analysis is not based on instantaneous properties of the signals, but it requires a segment to be computed, providing robustness in high variable arrhythmias like AF [14]. This robustness in inverse computed electrograms has been verified using both clinical and in-silico data. We have shown that DF patterns computed from electro-anatomical mapping are consistent with estimated iEGMs and allow for identifying DF gradients [30]. We also found that DF maps were reconstructed more accurately than phase and voltage maps. Later, we used 30 different AF simulations to quantify the robustness of High Dominant Frequency (HDF) maps against different uncertainty sources like atria location, orientation, or electrical noise [31]. HDF mapping resulted in a robust parameter with matching percentages of 73% ± 23% for 10-dB noise, 77% ± 21% for 5-cm displacement, and 60% ± 22% for 36° rotation.

The results obtained in the mentioned studies make the dominant frequency analysis of iEGMs a promising tool to characterize AF in both paroxysmal and persistent patients. However, there are still some limitations to overcome before reaching direct identification of AF drivers in DF maps. Firstly, the current standard method to obtain DFs consists in calculating the maximum spectral density of the signals [41, 70, 108]. Figure 3C depicts a comparison of the dominant frequency of intracardiac and ECGI maps and the correlation between intracardiac and noninvasive dominant frequencies [41]. This naïve approach does not prevent the identification of harmonic frequencies as DFs. Another important limitation, primarily present in persistent AF, is the variation of the frequency content through time, leading to the identification of irrelevant spectral components. This limitation has also been observed in clinics. In this regard, Sanders et. al. reported an increase in ablation time at DF sites, as well as a significant failure of AF termination during ablation (0 out of 13) in persistent with respect to paroxysmal AF [111]. A possible explanation for this is the mentioned lack of spatiotemporal stability of these sites, also reported in other studies [112, 113]. Therefore, according to the results obtained by these studies, additional analysis of the variation in the frequency domain may be relevant before applying any particular DF estimator to the signals.

## Machine learning in ECGI

The accuracy of current ECGI solutions is still suboptimal. The main reasons for this are some of the simplifications involved in the calculations, like treating the problem as a set of linear equations or the ill-posedness of the system to solve. These difficulties justify the use of more complex non-linear optimization techniques like machine learning.

A direct application of machine learning in ECGI would involve the estimation of a set of atrial electrical sources or an activation time distribution from noninvasive surface data. In this line, neural nets are a popular tool that has been tested using in-silico data with promising results. Karoui et. al. developed a data-driven approach called DirectMap in which activation times were directly estimated from BSPM recordings [114]. Later, the same authors proposed a spatial adaptation of the Time-Delay Neural Network (SATDNN-AT) to estimate activation times encoding the local dynamic propagation properties of the electrical activity [115]. In [116], a direct quantitative comparison between the two mentioned machine learning approaches with the classic technique of FEM combined with L1-norm regularization showed a clear outperformance of DirectMAP over the other two methods, and more importantly, that both machine learning approaches were strongly robust against additive gaussian noise compared to the traditional FEM method. Although this study could demonstrate the outperformance of DirectMAP over the traditional regularization method, this technique was only tested in in-silico data for a dataset containing single-paced rhythms. Therefore, further evaluation needs to be done in more complex rhythms like atrial fibrillation or clinical data. Apart from neural networks, other approaches have also been proven successful for activation time estimation. In [117], a regression algorithm (Reproducing Kernel Hilbert Space) was trained to estimate epicardial atrial potentials from simulated BSPM data. Although the EGM signals provided by the regression were not very accurate in terms of amplitude, this method would allow for an accurate estimation of the activation times. In order to improve the results in terms of voltage amplitude, the authors included the EGMs estimated by this regression model into a least squared regularization in a later study [118]. The results obtained from simulated electrical propagation patterns in the atria confirmed that their new regularization method based on the regression model increases the accuracy of the computed EGM with respect to the standard 0-order Tikhonov regularization. Machine learning algorithms can also be used to estimate non-invasively other relevant parameters of electrophysiological propagation models [119]. Some examples are activation onset location or tissue conductivity [120].

Besides finding the distribution of electrical sources or relevant electrophysiological parameters, machine learning has also been used to identify non-invasively other relevant arrhythmogenic phenomena in the atria, although most of the studies centered on this purpose used either ECG or BSPM signals. As an example, in [121], ECG signals from computer models were used to predict the locations of the AF drivers, reaching sensitivity and specificity levels of 73.9% and 82.6%, respectively. Specifically, the classifier was able to determine whether the AF drivers were located in the pulmonary vein areas or outside. This was done using a decision tree classifier with an in-silico data set for training and a set of 46 clinical ECGs for validation. Similar works in the same direction are, for example, [122], where the target was the location of ectopic foci using ECG signals in supraventricular tachycardias or the study of the inducibility in paroxysmal AF simulations including focal sources (ectopic foci and reentries) [123]. In this last study, features like the cycle length and periodicity of the simulated BSPM were used as features for a random forest classifier to identify inducibility, but also other parameters like the presence of focal sites in the atria. Regarding the location of focal drivers, J. Godoy et al. demonstrated the feasibility of the location of the origin in simulated focal atrial tachyarrhythmias with different degrees of fibrosis [124].

Besides specific mechanisms and complex electrophysiological data, many machine learning algorithms have also been developed for the automatic detection of atrial arrhythmias on the ECG [125, 126]. Although most of these works have not been tested for solving the inverse problem of electrocardiography, this technology appears very promising in this context as well.

## What is next?

ECGI allows both atria to be mapped synchronously, even with a single atrial beat, making it the first mapping technology of its kind. However, this panoramic view is gained at the expense of a lower spatial resolution as compared to invasive mapping techniques. Further research is required in order to properly identify the limitations of ECGI in the context of atrial arrhythmias.

**Fig. 5:**
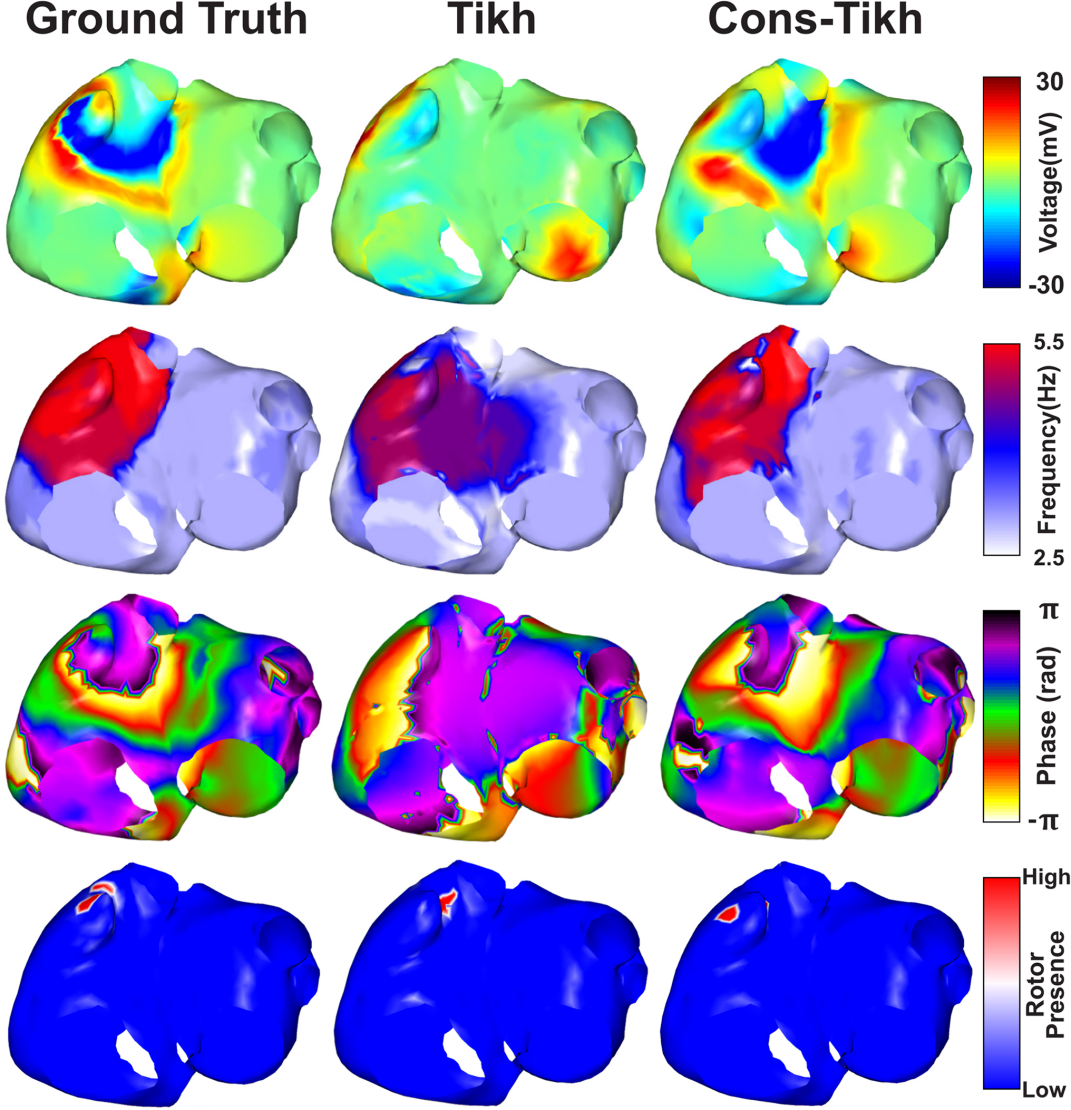
Comparison of reconstructions (epicardial potentials, dominant frequencies, phases, and phase singularities) between L0 Tikhonov and a new constrained Tikhonov regularization technique that combines noninvasive and invasive recordings, adapted from [82]

Regularization techniques have been more extensively validated in the context of ventricular signals and may not be entirely suitable for atrial signals. In particular, constraints typically imposed in order to choose both the regularization technique and the regularization parameters are not optimized for complex electrical patterns nor the presence of low signal-to-noise ratios. For this reason, regularization is possibly the most active area of research and dispute in the field of inverse problems. In this direction, we have recently proposed a new regularization technique that combines noninvasive and invasive recordings, illustrated in Fig. 5 [82]. This approach may overcome some of the limitations of both invasive and noninvasive atrial characterization and lead to a more accurate identification of AF drivers.

Recent studies have found significant line-of-block artifacts when computing activation maps from reconstructed ventricular potentials [90]. Although this has not been extensively studied in atrial signals [80], similar behavior can potentially be present in the estimation of atrial activation maps and should be further addressed since the main reason underlying these artifacts is spatial smoothing caused by regularization, which may be even more prominent in atrial reconstructions [127].

The lack of integration of structural abnormalities, such as scar, fibrosis, and other structural abnormalities, which are commonly encountered during atrial arrhythmias and have a significant impact on electrical patterns, is a significant limitation of current ECGI formulations. In this regard, scar-dependent ECGI solutions with ventricular data are starting to be proposed [128].

According to several experimental reports, ECGI algorithms based on the reconstruction of activation times may be more robust than those estimating the full course of potentials [129]. However, this particular ECGI resolution approach may be unable to deal effectively with reentrant events. A more exhaustive validation of activation imaging during fibrillatory rhythms and AF would be desirable. Another area where enhanced ECGI solutions can help is in the context of digital twins. Studies have been proposed for the generation of digital cardiac electrophysiology twins using 12-lead clinical ECGs [130], and ECGI could provide an extended set of restrictions to personalize computational models.

A promising field of research resides in the use of artificial intelligence to estimate the electrical activity of the heart. The use of autoencoders [131] and other machine learning architectures could be the future solution to the ill-posedness of the inverse problem.

Beyond these improvements in the estimation of cardiac potentials, further areas of research that should be further developed include the maturation of imageless technologies that will not require the co-registration of medical images to estimate the heart and torso geometries [13, 41]. In this context, machine learning approaches could potentially reduce the time for collecting the data needed for the ECGI calculation. Moreover, these methodologies could be used for the correct quantification of the real approximation of the ECGI solution, giving information on the confidence of the results for guiding and recommending treatments [132] and using them for the prediction of the evolution of cardiac arrhythmias [131].

Another important limitation of ECGI applied to surface signals is that we can only reconstruct epicardial activity. Similarly, non-contact catheter mapping only looks at endocardial tissue. Some work has attempted a combination of the two approaches in the ventricles [133, 134]. Future work on this pathway may help to unmask the phenomena of epi-endo dissociation in atria.

Finally, ECGI has the potential to guide ablation therapies in a totally noninvasive manner. While ablation today is performed by the introduction of catheters in the heart chambers and, therefore, the use of additional catheters for an electro-anatomical mapping does not introduce additional risks for the patient, in the future, the paradigm might change by further development of noninvasive ablation techniques such as stereotactic body radiation therapy (SBRT). SBRT consists of administering radiation therapy to a defined target with minimum collateral harm to surrounding tissue and has been successfully applied to treat ventricular arrhythmias [135]. Further improvements in spatial resolution on both ECGI and SBRT, together with validation studies in the context of atrial arrhythmias, may lead to a change in the current paradigm of treating atrial arrhythmias.

## Conclusion

The use of electrocardiographic imaging has the potential to reveal atrial arrhythmic substrates. The numerous approaches, technical aspects, and new solutions proposed in recent years in this field have highlighted the advances and existing difficulties for these techniques to be adopted by daily clinical practice in atrial mapping. However, these open issues can be considered an opportunity to overcome the existing limitations. Future ECGI studies and new research directions should help to fully elucidate the characterization and understanding of atrial arrhythmic mechanisms along with other imaging modalities for cross-validations. Enhancements in global atrial mapping techniques are essential to improve treatment guidance and patient outcomes.

## References

[1] Brugada J, Katritsis DG, Arbelo E (2020). 2019 ESC Guidelines for the management of patients with supraventricular tachycardiaThe Task Force for the management of patients with supraventricular tachycardia of the European Society of Cardiology (ESC): Developed in collaboration with the Association for European Paediatric and Congenital Cardiology (AEPC). Eur Heart J.

[2] König S, Ueberham L, Schuler E (2018). In-hospital mortality of patients with atrial arrhythmias: insights from the German-wide Helios hospital network of 161 502 patients and 34 025 arrhythmia-related procedures. Eur Heart J.

[3] Piccini JP, Hammill BG, Sinner MF (2012). Incidence and prevalence of atrial fibrillation and associated mortality among Medicare beneficiaries, 1993–2007. Circ Cardiovasc Qual Outcomes.

[4] Khurshid S, Choi SH, Weng L-C (2018). Frequency of cardiac rhythm abnormalities in a half million adults. Circ: Arrhythmia Electrophysiol.

[5] Lau DH, Nattel S, Kalman JM, Sanders P (2017). Modifiable risk factors and atrial fibrillation. Circulation.

[6] Page RL, Joglar JA, Caldwell MA (2016). 2015 ACC/AHA/HRS Guideline for the management of adult patients with supraventricular tachycardia: executive summary. Circulation.

[7] Bun S-S, Latcu DG, Delassi T (2016). Ultra-high-definition mapping of atrial arrhythmias. Circ J.

[8] Lee G, Sanders P, Kalman JM (2012). Catheter ablation of atrial arrhythmias: state of the art. The Lancet.

[9] Brignole M, Alboni P, Benditt DG (2004). Guidelines on management (diagnosis and treatment) of syncope–update 2004. Europace.

[10] Lemery R (2002). Bi-atrial mapping of atrial arrhythmias. Card Electrophysiol Rev.

[11] Shah A, Hocini M, Haissaguerre M, Jaïs P (2015). Non-invasive mapping of cardiac arrhythmias. Curr Cardiol Rep.

[12] Pereira H, Niederer S, Rinaldi CA (2020). Electrocardiographic imaging for cardiac arrhythmias and resynchronization therapy. Europace.

[13] Cluitmans M, Brooks DH, MacLeod R et al (2018) Validation and Opportunities of electrocardiographic imaging: from technical achievements to clinical applications. Front Physiol 9:1305. 10.3389/fphys.2018.01305 10.3389/fphys.2018.01305 PMC6158556 30294281

[14] Salinet J, Molero R, Schlindwein FS (2021). Electrocardiographic imaging for atrial fibrillation: a perspective from computer models and animal experiments to clinical value. Front Physiol.

[15] January CT, Wann LS, Alpert JS (2014). 2014 AHA/ACC/HRS guideline for the management of patients with atrial fibrillation: executive summary. Circulation.

[16] Goodacre S, Irons R (2002). ABC of clinical electrocardiography: Atrial arrhythmias. BMJ.

[17] Jalife J (2011). Déjà vu in the theories of atrial fibrillation dynamics. Cardiovasc Res.

[18] Guillem MS, Climent AM, Rodrigo M (2016). Presence and stability of rotors in atrial fibrillation: evidence and therapeutic implications. Cardiovasc Res.

[19] Nattel S, Dobrev D (2017). Controversies about atrial fibrillation mechanisms. Circ Res.

[20] Jaïs P, Haïssaguerre M, Shah DC (1997). A focal source of atrial fibrillation treated by discrete radiofrequency ablation. Circulation.

[21] Jalife J, Berenfeld O, Mansour M (2002). Mother rotors and fibrillatory conduction: a mechanism of atrial fibrillation. Cardiovasc Res.

[22] Gk M (1962). On the multiple wavelet hypothesis of atrial fibrillation. Arch Int Pharmacodyn Ther.

[23] Haissaguerre M, Shah AJ, Cochet H (2016). Intermittent drivers anchoring to structural heterogeneities as a major pathophysiological mechanism of human persistent atrial fibrillation. J Physiol.

[24] van der Does LJME, Kik C, Bogers AJJC (2016). Dynamics of endo- and epicardial focal fibrillation waves at the right atrium in a patient with advanced atrial remodelling. Can J Cardiol.

[25] García-Cosío F, Pastor Fuentes A, Núñez Angulo A (2012). Clinical approach to atrial tachycardia and atrial flutter from an understanding of the mechanisms. Electrophysiology Based on Anatomy. Revista Española de Cardiología (English Edition).

[26] Cosío FG, Getafe University Hospital, European University of Madrid, Madrid, Spain (2017). Atrial flutter, typical and atypical: a review. Arrhythm Electrophysiol Rev.

[27] Bun S-S, Latcu DG, Marchlinski F, Saoudi N (2015). Atrial flutter: more than just one of a kind. Eur Heart J.

[28] Markowitz SM, Thomas G, Liu CF (2019). Atrial tachycardias and atypical atrial flutters: mechanisms and approaches to ablation. Arrhythm Electrophysiol Rev.

[29] Issa ZF, Miller JM, Zipes DP (2012) Focal atrial tachycardia. Clinical Arrhythmology and electrophysiology: A companion to Braunwald’s heart disease, 2nd edn. Elsevier Health Sciences, Philadelphia, pp 212–237

[30] Buttà C, Tuttolomondo A, Giarrusso L, Pinto A (2015). Electrocardiographic diagnosis of atrial tachycardia: classification, P-wave morphology, and differential diagnosis with other supraventricular tachycardias. Ann Noninvasive Electrocardiol.

[31] Custer AM, Yelamanchili VS, Lappin SL (2021) Multifocal atrial tachycardia. StatPearls Publishing, Treasure Island 29083603

[32] Olshansky B, Sullivan RM (2013). Inappropriate sinus tachycardia. J Am Coll Cardiol.

[33] Hafeez Y, Grossman SA (2019) Sinoatrial nodal reentrant tachycardia. StatPearls Publishing, Treasure Island 29939564

[34] Rudy Y, Messinger-Rapport BJ (1988). The inverse problem in electrocardiography: solutions in terms of epicardial potentials. Crit Rev Biomed Eng.

[35] Pullan AJ, Cheng LK, Nash MP, Macfarlane PW, van Oosterom A, Pahlm O (2010). The inverse problem of electrocardiography. Comprehensive electrocardiology.

[36] Brooks DH, Ahmad GF, MacLeod RS, Maratos GM (1999). Inverse electrocardiography by simultaneous imposition of multiple constraints. IEEE Trans Biomed Eng.

[37] Modre R, Tilg B, Fischer G, Wach P (2002). Noninvasive myocardial activation time imaging: a novel inverse algorithm applied to clinical ECG mapping data. IEEE Trans Biomed Eng.

[38] Franzone PC, Taccardi B, Viganotti C (1978). An approach to inverse calculation of epicardial potentials from body surface maps. Adv Cardiol.

[39] Rudy Y (2017). Noninvasive ECG imaging (ECGI): Mapping the arrhythmic substrate of the human heart. Int J Cardiol.

[40] Kim D, Ahn H (2012) Current status and future of cardiac mapping in atrial fibrillation. Atrial Fibrillation‐Basic Research and Clinical Applications. Intech, Rijeka, pp 108–117

[41] Rodrigo M, Waddell K, Magee S et al (2021) Non-invasive spatial mapping of frequencies in atrial fibrillation: correlation with contact mapping. Front Physiol 11:611266. 10.3389/fphys.2020.611266 10.3389/fphys.2020.611266 PMC7873897 33584334

[42] Grace A, Willems S, Meyer C (2019). High-resolution noncontact charge-density mapping of endocardial activation. JCI Insight.

[43] Shi R, Parikh P, Chen Z (2020). Validation of dipole density mapping during atrial fibrillation and sinus rhythm in human left atrium. JACC: Clin Electrophysiol.

[44] Shi R, Chen Z, Butcher C (2020). Diverse activation patterns during persistent atrial fibrillation by noncontact charge-density mapping of human atrium. J Arrhythmia.

[45] Ramak R, Chierchia G-B, Paparella G (2021). Novel noncontact charge density map in the setting of post-atrial fibrillation atrial tachycardias: first experience with the Acutus SuperMap Algorithm. J Interv Card Electrophysiol.

[46] Cuculich PS, Wang Y, Lindsay BD (2010). Noninvasive characterization of epicardial activation in humans with diverse atrial fibrillation patterns. Circulation.

[47] Haissaguerre M, Hocini M, Denis A (2014). Driver domains in persistent atrial fibrillation. Circulation.

[48] MacLeod R, Buist M, Macfarlane PW, van Oosterom A, Pahlm O (2010). The forward problem of electrocardiography. Comprehensive electrocardiology.

[49] Horácek BM, Clements JC (1997). The inverse problem of electrocardiography: a solution in terms of single- and double-layer sources of the epicardial surface. Math Biosci.

[50] Barr RC, Ramsey M, Spach MS (1977). Relating epicardial to body surface potential distributions by means of transfer coefficients based on geometry measurements. IEEE Trans Biomed Eng.

[51] Wang D, Kirby RM, Johnson CR (2010). Resolution strategies for the finite-element-based solution of the ECG inverse problem. IEEE Trans Biomed Eng.

[52] Wang Y, Rudy Y (2006). Application of the method of fundamental solutions to potential-based inverse electrocardiography. Ann Biomed Eng.

[53] Fischer G, Tilg B, Wach P (1999). Application of high-order boundary elements to the electrocardiographic inverse problem. Comput Methods Programs Biomed.

[54] Wang D, Kirby RM, Johnson CR (2011). Finite-element-based discretization and regularization strategies for 3-D inverse electrocardiography. IEEE Trans Biomed Eng.

[55] Pilkington TC, Morrow MN, Stanley PC (1985). A comparison of finite element and integral equation formulations for the calculation of electrocardiographic potentials. IEEE Trans Biomed Eng.

[56] Pilkington TC, Morrow MN, Stanley PC (1987). A comparison of finite element and integral equation formulations for the calculation of electrocardiographic potentials–II. IEEE Trans Biomed Eng.

[57] Seger M, Fischer G, Modre R (2005). Lead field computation for the electrocardiographic inverse problem—finite elements versus boundary elements. Comput Methods Programs Biomed.

[58] Wang H, Qin Q-H, Wang H, Qin Q-H (2019). Chapter 1 - Overview of meshless methods. Methods of fundamental solutions in solid mechanics.

[59] Tikhonov AN, Arsenin VY (1977). Solutions of ill-posed problems.

[60] Calvetti D, Lewis B, Reichel L (2002). GMRES, L-curves, and discrete ill-posed problems. BIT Numer Math.

[61] Figuera C, Suárez-Gutiérrez V, Hernández-Romero I et al (2016) Regularization techniques for ECG imaging during atrial fibrillation: a computational study. Front Physiol 7:466. 10.3389/fphys.2016.00466 10.3389/fphys.2016.00466 PMC5064166 27790158

[62] van Oosterom A, van Dam P (2005). The intra-myocardial distance function used in inverse computations of the timing of depolarization and repolarization. Comput Cardiol.

[63] Borràs M, Chamorro-Servent J (2021). Electrocardiographic imaging: a comparison of iterative solvers. Front Physiol.

[64] Ramanathan C, Jia P, Ghanem R (2003). Noninvasive electrocardiographic imaging (ECGI): application of the generalized minimal residual (GMRes) method. Ann Biomed Eng.

[65] Messinger-Rapport BJ, Rudy Y (1990). Noninvasive recovery of epicardial potentials in a realistic heart-torso geometry Normal sinus rhythm. Circu Res.

[66] Hansen PC (1992). Analysis of discrete ill-posed problems by means of the L-curve. SIAM Rev.

[67] Hansen PC, O’Leary DP (1993). The use of the L-curve in the regularization of discrete ill-posed problems. SIAM J Sci Comput.

[68] Colli-Franzone P, Guerri L, Tentoni S (1985). A mathematical procedure for solving the inverse potential problem of electrocardiography. analysis of the time-space accuracy from in vitro experimental data. Math Biosci.

[69] Pedrón-Torrecilla J, Rodrigo M, Climent AM (2016). Noninvasive estimation of epicardial dominant high-frequency regions during atrial fibrillation. J Cardiovasc Electrophysiol.

[70] Rodrigo M, Climent AM, Liberos A (2017). Highest dominant frequency and rotor positions are robust markers of driver location during noninvasive mapping of atrial fibrillation: a computational study. Heart Rhythm.

[71] Rodrigo M, Climent AM, Hernández-Romero I (2020). Noninvasive assessment of complexity of atrial fibrillation. Circ: Arrhythmia Electrophysiol.

[72] Rodrigo M, Climent AM, Liberos A (2018). Solving inaccuracies in anatomical models for electrocardiographic inverse problem resolution by maximizing reconstruction quality. IEEE Trans Med Imaging.

[73] Shah AJ, Hocini M, Pascale P (2013). Body surface electrocardiographic mapping for non-invasive identification of arrhythmic sources. Arrhythm Electrophysiol Rev.

[74] Gao X, Lam AG, Bilchick KC (2019). The use of non-invasive mapping in persistent AF to predict acute procedural outcome. J Electrocardiol.

[75] Shah AJ, Hocini M, Xhaet O (2013). Validation of novel 3-dimensional electrocardiographic mapping of atrial tachycardias by invasive mapping and ablation. J Am Coll Cardiol.

[76] Cheniti G, Puyo S, Martin CA (2019). Noninvasive mapping and electrocardiographic imaging in atrial and ventricular arrhythmias (CardioInsight). Card Electrophysiol Clin.

[77] Wang Y, Cuculich PS, Woodard PK (2007). Focal atrial tachycardia after pulmonary vein isolation: noninvasive mapping with electrocardiographic imaging (ECGI). Heart Rhythm.

[78] Wang Y, Rudy Y (2009). Electrocardiographic imaging of normal human atrial repolarization. Heart Rhythm.

[79] Ramanathan C, Ghanem RN, Jia P (2004). Noninvasive electrocardiographic imaging for cardiac electrophysiology and arrhythmia. Nat Med.

[80] Schill MR, Cuculich PS, Andrews CM (2020). The arrhythmic substrate for atrial fibrillation in patients with mitral regurgitation. J Atr Fibrillation.

[81] Serinagaoglu Y, Brooks DH, MacLeod RS (2005). Bayesian solutions and performance analysis in bioelectric inverse problems. IEEE Trans Biomed Eng.

[82] Cámara-Vázquez MÁ, Hernández-Romero I, Rodrigo M (2021). Electrocardiographic imaging including intracardiac information to achieve accurate global mapping during atrial fibrillation. Biomed Signal Process Control.

[83] Milanič M, Jazbinšek V, MacLeod RS (2014). Assessment of regularization techniques for electrocardiographic imaging. J Electrocardiol.

[84] Chamorro-Servent J, Dubois R, Coudière Y (2019) Considering new regularization parameter-choice techniques for the Tikhonov method to improve the accuracy of electrocardiographic imaging. Front Physiol 10:273. 10.3389/fphys.2019.00273 10.3389/fphys.2019.00273 PMC6445955 30971937

[85] Modre R, Tilg B, Fischer G (2003). Atrial noninvasive activation mapping of paced rhythm data. J Cardiovasc Electrophysiol.

[86] Seger M, Modre R, Pfeifer B et al (2006) Non-invasive imaging of atrial flutter. Comput cardiol 2006:601−604

[87] Scharf G, Dang L (2016) Dipole density instead of potentials in electrocardiology. arXiv preprint. https://doi.org/10.48550/arXiv.1601.04419

[88] Cantwell CD, Roney CH, Ng FS (2015). Techniques for automated local activation time annotation and conduction velocity estimation in cardiac mapping. Comput Biol Med.

[89] Zhou Z, Jin Q, Yu L (2016). Noninvasive imaging of human atrial activation during atrial flutter and normal rhythm from body surface potential maps. PLoS ONE.

[90] Bear LR, Bouhamama O, Cluitmans M (2019). Advantages and pitfalls of noninvasive electrocardiographic imaging. J Electrocardiol.

[91] Cluitmans M, Coll-Font J, Erem B (2021). Spatiotemporal approximation of cardiac activation and recovery isochrones. J Electrocardiol.

[92] Schaufelberger M, Schuler S, Bear L, et al (2019) Comparison of activation times estimation for potential-based ECG imaging. Comput cardiol 2019:1-4. https://doi.org/10.22489/CinC.2019.379 10.22489/cinc.2019.379 PMC7079739 32190705

[93] Duchateau J, Potse M, Dubois R (2017). Spatially coherent activation maps for electrocardiographic imaging. IEEE Trans Biomed Eng.

[94] Vijayakumar R, Vasireddi SK, Cuculich PS (2016). Methodology considerations in phase mapping of human cardiac arrhythmias. Circ: Arrhythmia Electrophysiol.

[95] Liberos A, Rodrigo M, Hernandez-Romero I (2019). Phase singularity point tracking for the identification of typical and atypical flutter patients: a clinical-computational study. Comput Biol Med.

[96] Bray M-A, Wikswo JP (2002). Use of topological charge to determine filament location and dynamics in a numerical model of scroll wave activity. IEEE Trans Biomed Eng.

[97] Iyer AN, Gray RA (2001). An experimentalist’s approach to accurate localization of phase singularities during reentry. Ann Biomed Eng.

[98] Cizek V (1970). Discrete Hilbert transform.

[99] Li X, Almeida TP, Dastagir N et al (2020) Standardizing single-frame phase singularity identification algorithms and parameters in phase mapping during human atrial fibrillation. Front Physiol 11:869. 10.3389/fphys.2020.00869 10.3389/fphys.2020.00869 PMC7386053 32792983

[100] Rodrigo M, Climent AM, Liberos A (2017). Technical considerations on phase mapping for identification of atrial reentrant activity in direct- and inverse-computed electrograms. Circ: Arrhythmia Electrophysiol.

[101] Podziemski P, Zeemering S, Kuklik P (2018). Rotors detected by phase analysis of filtered, epicardial atrial fibrillation electrograms colocalize with regions of conduction block. Circ: Arrhythmia Electrophysiol.

[102] Van Nieuwenhuyse E, Martinez-Mateu L, Saiz J (2021). Directed graph mapping exceeds phase mapping in discriminating true and false rotors detected with a basket catheter in a complex in-silico excitation pattern. Comput Biol Med.

[103] Martinez-Mateu L, Romero L, Ferrer-Albero A (2018). Factors affecting basket catheter detection of real and phantom rotors in the atria: a computational study. PLoS Comput Biol.

[104] Rodrigo M, Guillem MS, Climent AM (2014). Body surface localization of left and right atrial high-frequency rotors in atrial fibrillation patients: a clinical-computational study. Heart Rhythm.

[105] Bear LR, Dogrusoz YS, Good W (2021). The impact of torso signal processing on noninvasive electrocardiographic imaging reconstructions. IEEE Trans Biomed Eng.

[106] Hansen BJ, Zhao J, Csepe TA (2015). Atrial fibrillation driven by micro-anatomic intramural re-entry revealed by simultaneous sub-epicardial and sub-endocardial optical mapping in explanted human hearts. Eur Heart J.

[107] Atienza F, Almendral J, Jalife J (2009). Real-time dominant frequency mapping and ablation of dominant frequency sites in atrial fibrillation with left-to-right frequency gradients predicts long-term maintenance of sinus rhythm. Heart Rhythm.

[108] Guillem MS, Climent AM, Millet J (2013). Noninvasive localization of maximal frequency sites of atrial fibrillation by body surface potential mapping. Circ: Arrhythmia Electrophysiol.

[109] Di Biase L, Burkhardt JD, Mohanty P (2010). Left atrial appendage. Circulation.

[110] Hocini M, Nault I, Wright M (2010). Disparate evolution of right and left atrial rate during ablation of long-lasting persistent atrial fibrillation. J Am Coll Cardiol.

[111] Sanders P, Berenfeld O, Hocini M (2005). Spectral analysis identifies sites of high-frequency activity maintaining atrial fibrillation in humans. Circulation.

[112] Sih HJ, Zipes DP, Berbari EJ (2000). Differences in organization between acute and chronic atrial fibrillation in dogs. J Am Coll Cardiol.

[113] Akar JG, Everett TH, Kok L-C (2002). Effect of electrical and structural remodeling on spatiotemporal organization in acute and persistent atrial fibrillation. J Cardiovasc Electrophysiol.

[114] Karoui A, Bendahmane M, Zemzemi N (2019) Direct mapping from body surface potentials to cardiac activation maps using neural networks. Comput cardiol 2019:1–4. 10.22489/CinC.2019.253

[115] Karoui A, Bendahmane M, Zemzemi N, Coudière Y, Ozenne V, Vigmond E, Zemzemi N (2019). A spatial adaptation of the time delay neural network for solving ECGI inverse problem. Functional imaging and modeling of the heart.

[116] Karoui A, Bendahmane M, Zemzemi N (2021) Cardiac activation maps reconstruction: a comparative study between data-driven and physics-based methods. Front Physiol 12:1265. 10.3389/fphys.2021.686136 10.3389/fphys.2021.686136 PMC8428526 34512373

[117] Zemzemi N, Labarthe S, Dubois R, Coudière Y (2012) From body surface potential to activation maps on the atria: a machine learning technique. Comput cardiol 2012:125−128

[118] Zemzemi N, Dubois R, Coudière Y (2013). A machine learning regularization of the inverse problem in electrocardiography imaging. Comput cardiol.

[119] Coll-Font J, Wang L, Brooks DH (2018) A common-ground review of the potential for machine learning approaches in electrocardiographic imaging based on probabilistic graphical models. Comput cardiol 2018:1-4. 10.22489/CinC.2018.348 10.22489/CinC.2018.348 PMC6424344 30899763

[120] Giffard-Roisin S, Jackson T, Fovargue L (2017). Noninvasive personalization of a cardiac electrophysiology model from body surface potential mapping. IEEE Trans Biomed Eng.

[121] Luongo G, Azzolin L, Rivolta MW, et al (2020) Non-Invasive identification of atrial fibrillation driver location using the 12-lead ECG: pulmonary vein rotors vs. other locations. 42nd Annual International Conference of the IEEE Engineering in Medicine Biology Society 2020:410–413. 10.1109/EMBC44109.2020.9176135 10.1109/EMBC44109.2020.9176135 33018015

[122] Mohammadi F, Sheikhani A, Razzazi F, Ghorbani Sharif A (2021). Non-invasive localization of the ectopic foci of focal atrial tachycardia by using ECG signal based sparse decomposition algorithm. Biomed Signal Process Control.

[123] Feng Y, Roney CH, Bayer JD (2022). Detection of focal source and arrhythmogenic substrate from body surface potentials to guide atrial fibrillation ablation. PLoS Comput Biol.

[124] Godoy EJ, Lozano M, García-Fernández I (2018). Atrial fibrosis hampers non-invasive localization of atrial ectopic foci from multi-electrode signals: a 3D simulation study. Front Physiol.

[125] Król-Józaga B (2022). Atrial fibrillation detection using convolutional neural networks on 2-dimensional representation of ECG signal. Biomed Signal Process Control.

[126] Tseng C-W, Lin G-H, Chang C-H, Jobbágy Á (2012). Automatic detection of atrial fibrillation based on handheld ECG device. 5th European Conference of the International Federation for Medical and Biological Engineering.

[127] Schuler S, Schaufelberger M, Bear LR et al (2021) Reducing line-of-block artifacts in cardiac activation maps estimated using ECG imaging: a comparison of source models and estimation methods. IEEE Trans Biomed Eng 69(6):2041-2052. 10.1109/TBME.2021.3135154 10.1109/TBME.2021.3135154 34905487

[128] Diallo MM, Potse M, Dubois R, Coudiére Y (2020) Solving the ECGI problem with known locations of scar regions. Comput cardiol 2020:1–4. 10.22489/CinC.2020.237

[129] Oosterhoff P, Meijborg VMF, van Dam PM (2016). Experimental validation of noninvasive epicardial and endocardial activation imaging. Circ: Arrhythmia Electrophysiol.

[130] Gillette K, Gsell MAF, Prassl AJ (2021). A framework for the generation of digital twins of cardiac electrophysiology from clinical 12-leads ECGs. Med Image Anal.

[131] Bacoyannis T, Ly B, Cedilnik N (2021). Deep learning formulation of electrocardiographic imaging integrating image and signal information with data-driven regularization. EP Europace.

[132] Molero R, Soler Torro JM, Martínez Alzamora N (2021). Higher reproducibility of phase derived metrics from electrocardiographic imaging during atrial fibrillation in patients remaining in sinus rhythm after pulmonary vein isolation. Comput Biol Med.

[133] Revishvili AS, Wissner E, Lebedev DS (2015). Validation of the mapping accuracy of a novel non-invasive epicardial and endocardial electrophysiology system. EP Europace.

[134] Good WW, Zenger B, Bergquist JA (2021). Combining endocardial mapping and electrocardiographic imaging (ECGI) for improving PVC localization: a feasibility study. J Electrocardiol.

[135] Benedict SH, Yenice KM, Followill D (2010). Stereotactic body radiation therapy: the report of AAPM task group 101. Med Phys.

